# A randomized controlled trial of postoperative intravenous acetaminophen plus thoracic epidural analgesia vs. thoracic epidural analgesia alone after gastrectomy for gastric cancer

**DOI:** 10.1007/s10120-018-0863-5

**Published:** 2018-08-07

**Authors:** Jun Kinoshita, Sachio Fushida, Masahide Kaji, Katsunobu Oyama, Daisuke Fujimoto, Yasuo Hirono, Tomoya Tsukada, Takashi Fujimura, Shigekazu Ohyama, Kazuhisa Yabushita, Naotaka Kadoya, Koji Nishijima, Tetsuo Ohta, On behalf of the Digestive Disease Support Organization Study Group, Sachio Fushida, Jun Kinoshita, Katsunobu Oyama, Takashi Fujimura, Naotaka Kadoya, Toru Li, Seiichi Yamamoto, Masahide Kaji, Tomoya Tsukada, Kazuhisa Yabushita, Nariatsu Sato, Tatsuo Nakano, Toshiaki YasuiShigekazu Ohyama, Koji Nishijima, Takashi Tani, Yutaka Yoshimitsu, Shinichi Kinami, Daisuke Fujimoto, Yasuo Hirono

**Affiliations:** 1 Department of Gastroenterological Surgery, Kanazawa University Hospital, 13-1 Takara-machi, Kanazawa, 920-8641 Japan; 2 Department of Surgery, Toyama Prefectural Central Hospital, Toyama, Japan; 3 First Department of Surgery, Fukui University Hospital, Fukui, Japan; 4 Department of Surgery, Toyama City Hospital, Toyama, Japan; 5 Department of Surgery, National Hospital Organization Kanazawa Medical Center, Kanazawa, Japan; 6 Department of Surgery, Takaoka City Hospital, Takaoka, Japan; 7 Department of Surgery, Toyama Rosai Hospital, Uozu, Japan; 8 Department of Surgery, Japanese Red Cross Kanazawa Hospital, Kanazawa, Japan

**Keywords:** Acetaminophen, Multimodal analgesia, Gastrectomy, Gastric cancer

## Abstract

**Background:**

Acetaminophen is used in multimodal therapy for postoperative pain management. However, the additional effects of acetaminophen in combination with thoracic epidural analgesia (TEA) are not well understood. This prospective, multicenter randomized study was conducted to evaluate the efficacy of routine intravenous (i.v.) acetaminophen in combination with TEA for the management of postoperative pain in gastric cancer surgery.

**Methods:**

A total of 120 patients who underwent distal gastrectomy were randomly assigned in a 1:1 ratio to receive i.v. acetaminophen every 6 h and TEA during the first 3 postoperative days (acetaminophen group) or TEA alone (control group). The primary endpoint was the sum of TEA rescue doses during the first 2 postoperative days.

**Results:**

Final analysis included 58 patients in the acetaminophen group and 56 patients in the control group. The median number of TEA rescue doses was significantly lower in the acetaminophen group compared with the control group (3.0 vs. 8.0, *p* = 0.013). The median area under the curve (AUC) of the pain scores at coughing was significantly less in the acetaminophen group compared with the control group (285 vs. 342, *p* = 0.046) without an increase in postoperative complications. TEA rescue doses and pain score AUCs were significantly reduced by acetaminophen in patients who underwent open gastrectomy (*p* = 0.037 and 0.045), whereas there was no significant difference between patients who underwent laparoscopic gastrectomy in the two groups.

**Conclusions:**

In gastric cancer surgery patients, routine i.v. acetaminophen in combination with TEA provides superior postoperative pain management compared with TEA alone.

## Introduction

Gastric cancer (GC) is the fifth most common cancer and the third leading cause of cancer-related deaths worldwide [1]. Although surgical techniques have greatly improved, GC surgery remains a high-risk procedure that is associated with various postoperative complications. The morbidity and mortality rates after radical gastrectomy have been reported to be 9.1–28.1 and 0–1.3%, respectively [2–6]. Pain management during perioperative periods is important to prevent complications involving the circulatory, respiratory, digestive, and nervous system, especially because postoperative pain following upper abdominal surgery, such as gastric surgery, is strongly influenced by the respiratory system. The enhanced recovery after surgery (ERAS) protocol has been increasingly used to alleviate postoperative pain and complications and to improve the patient’s early recovery capacity [7–9]. Pain, immobility, and decreased intestinal function have been cited as inhibitors of postoperative recovery, and adequate pain management is essential because pain can cause both of immobility and decreased intestinal function.

Thoracic epidural analgesia (TEA) is the gold standard for open abdominal surgery pain management, as described in an ERAS consensus statement for gastrointestinal surgery [10]. In Japan, TEA is generally used as perioperative analgesia during GC surgery. In a prospective study, analgesic effects of TEA were shown to be significantly stronger compared with postoperative pain in a patient who received intravenous analgesia for postoperative pain management after radical gastrectomy for GC [11].

Recently, multimodal analgesia (MMA), which is designed to enhance analgesic effects using combinations of analgesics with different mechanisms of action, has attracted attention. MMA reduces opioid consumption, minimizes adverse events, improves pain relief, facilitates earlier recovery, and reduces hospitalization costs [12, 13]. In the ERAS consensus statement, a MMA regimen based on routine use of NSAIDs, COX-2, and acetaminophen is recommended in open and laparoscopic abdominal procedures. Acetaminophen is now available for intravenous (i.v.) use, and it is considered to be a valuable component of MMA because, unlike NSAIDs, it does not have unwanted side effects such as intestinal bleeding or renal toxicity [10]. The key advantage of i.v. acetaminophen seems to be that i.v. acetaminophen has a concentration in plasma and organs that is twice as high as that observed after oral or rectal administration, resulting in greater central nervous system penetration [14]. Multiple prospective trials comparing i.v. acetaminophen with placebo in patients undergoing orthopedic, laparoscopic, and endoscopic sinus surgeries demonstrated that i.v. acetaminophen improves pain relief, has an opioid-sparing effect, increases patient satisfaction, and decreases the requirement for rescue medications [15, 16]. However, these clinical trials involved minimally invasive surgeries with moderate pain such as laparoscopic cholecystectomy or gynecologic surgery, and TEA was not used concurrently. Considering the strong analgesic effect of TEA, it is unclear whether the addition of i.v. acetaminophen with TEA for GC surgery provides the advantage of further pain relief. In laparoscopic gastrectomy, which has been increasingly used in recent years, it is questionable whether i.v. acetaminophen with TEA is necessary because the postoperative pain is mild compared to open gastrectomy.

To date, there has not been a prospective study focused on the effects of i.v. acetaminophen in combination with TEA for postoperative analgesia after gastrectomy. Therefore, we conducted a prospective, multicenter randomized study in patients undergoing laparoscopic or open distal gastrectomy for GC to assess the role of routine i.v. acetaminophen with TEA in reducing postoperative pain. The primary endpoint of the study focused on postoperative pain evaluated using the sum of TEA rescue doses during the first 2 postoperative days. We hypothesized that i.v. acetaminophen with TEA improves postoperative pain relief following gastrectomy.

## Materials and methods

### Patients and methods

This randomized, prospective, parallel-group superiority study was conducted at 18 sites in Japan between August 2016 and October 2017 to evaluate the efficacy and safety of i.v. acetaminophen in combination with TEA for the treatment of postoperative pain in patients undergoing radical distal gastrectomy for GC.

The study followed the principles of the Declaration of Helsinki and was approved by institutional review boards and independent ethics committees at all participating institutions. The trial was registered under UMIN (Trial no. 000022710) before patient recruitment was started.

Before participation, patients received information about the study, including details of the treatment procedure, and provided written informed consent. Inclusion criteria was as follows: patients with histologically proven adenocarcinoma of the stomach, tumor (cT1-4a, N0-3b, M0) that was surgically resectable by distal gastrectomy, and ECOG performance status 0 or 1. Patients were excluded if they were < 20 years old; unable to provide informed consent or a reliable self-report of pain; had a history of allergy or hypersensitivity to acetaminophen, aspirin, or NSAIDs; had impaired glucose tolerance or hypertension; were anemic; had a history of asthma, heart failure, or hepatic disease; were pregnant or breastfeeding; or had a medical contraindication for TEA based on institutional guidelines.

### Study setting

After consent was obtained from the patients, they were randomized in a 1:1 ratio to receive either the TEA alone (control group) or combination treatment with routine i.v. acetaminophen and TEA (acetaminophen group). Patients were randomly stratified by the type of surgery (open or laparoscopic gastrectomy) and institution. In both patient groups, an epidural catheter was inserted at thoracic level Th8–Th10 using standard techniques, in accordance with institutional practices before anesthesia induction.

According to the Japanese Gastric Cancer Treatment Guidelines [17], we performed laparoscopic or open distal gastrectomy with D1 + or D2 lymphadenectomy depending on the degree of progression and surgical risk. The reconstruction technique was either Billroth-I, II, or Roux-en Y. The choice of surgical procedure (open or laparoscopic) was left to the surgeon’s discretion.

At the end of each surgery, we obtained a radiograph to check the position of the epidural catheter. Postoperatively, epidural analgesia was maintained for 3 days with a silicone balloon infuser containing 10 mL fentanyl citrate and 290 mL 0.2% ropivacaine. The balloon pump infuser was set to 4 mL/h for continuous infusion, and it was supplemented by rescue boluses of 3 mL with a 60-min lock-out period. A rescue bolus of the epidural infusion was administrated if the numeric rating scale (NRS) at rest was greater than 3. The epidural catheter was removed at the end of the medical solution in the balloon pump infuser on postoperative day (POD) 3.

**Fig. 1:**
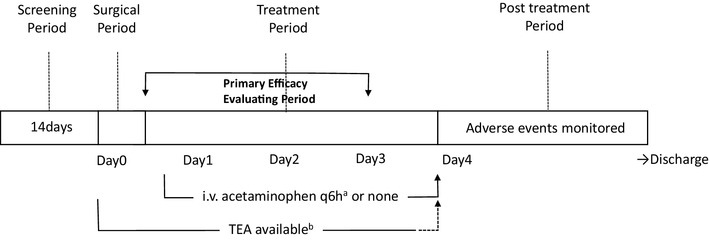
**a** i.v. acetaminophen was administered every 6 h from 6 h after surgery up to 72 h (3 days) in the acetaminophen group. **b***TEA* thoracic epidural analgesia. TEA was maintained for 3 postoperative days, then removed at the end of the medical solution in the balloon pump infuser

For patients randomized to the acetaminophen group, the first dose of i.v. acetaminophen (Acelio Intravenous Injection; Terumo Co. Ltd., Tokyo, Japan) was administered 6 h after completion of surgery, at a dose of 1000 mg/dose for patients weighting ≥ 50 kg or at 15 mg/kg/dose for patients weighting < 50 kg. Subsequent acetaminophen doses were administered every 6 h (the dose was infused over 15 min). The treatment period was the first 3 postoperative days (Fig. 1).

Predefined rescue medications were used for breakthrough pain that could not be controlled even using a rescue TEA bolus during the 3 postoperative days. The choice of analgesic was limited to acetaminophen, flurbiprofen axetil, or pentazocine hydrochloride in the control group, and to flurbiprofen axetil, or pentazocine hydrochloride in the acetaminophen group. Postoperative nausea and vomiting (PONV) was defined when it was sufficient to warrant treatment with one or more additional doses of metoclopramide.

### Study endpoints

The primary endpoint of this study was the difference in the sum of rescue TEA doses during the first 2 postoperative days in the acetaminophen and control groups.

Secondary endpoints included pain relief assessed using the 11-point NRS (0 = no pain to 10 = intense pain). Patients were familiarized with the scale preoperatively, and routine evaluation daily at rest and during coughing every 12 h began on the morning of POD1 and continued until POD3. To assess differences in the overall pain during the treatment period, the area under the NRS–pain curve (AUC) was calculated for the first 3 postoperative days (AUC72) using the trapezoidal method. Other secondary endpoints included fentanyl consumption during the first 2 postoperative days, the number of rescue medications use during the first 3 postoperative days, frequency of PONV, the recovery of bowel function (the time to first flatus and defecation), the first day of ambulation, and duration of postoperative hospital stay.

Postoperative 30-day morbidity and mortality were graded using the Clavien–Dindo classification system (CD) [18], with grade ≥ 2 events recorded as complications; severe complications were defined as complication grade 3–5. The tolerability and safety profile of the routine i.v. acetaminophen was also evaluated by adverse events reported from the first dose to discharge, and also by laboratory assessments, vital signs (i.e., blood pressure while semi-recumbent and heart rate), and physical examination results. Laboratory test results were monitored on POD1, 3, and 7 to investigate the renal, liver and hematology values. Postoperative liver dysfunction was graded according to the Common Terminology Criteria for Adverse Events, with grade ≥ 3 events (defined as ≥ 5-times the upper limit of normal) recorded as complications.

Demographic information (age, sex, body mass index, and the American Society of Anesthesiologists score) and pertinent surgical information (indication, type of surgery, surgical time, and estimated blood loss) were recorded.

### Statistical analysis

The sum of TEA rescue doses during the first 2 postoperative days was determined as the primary outcome. Sample size determination was based on the previous retrospective study on i.v. acetaminophen treatment after gastrectomy [19]. It was projected that the sum of TEA rescue doses would be reduced from 37% by the combined use of the routine i.v. acetaminophen. Taking a 35% decrease in the sum of TEA rescue doses as clinically significant, each group required 53 patients to detect a difference with type I error of 0.05 and a power of 0.8. To allow for dropouts, 60 patients were recruited for each group.

Descriptive statistics were reported as the absolute or relative frequencies for categorical variables and as the median (range or interquartile range [IQR]) or mean ± standard deviation for continuous variables as appropriate. Fisher’s exact test was used to analyze categorical variables. The Student’s *t* test and the Mann–Whitney *U* test were used to compare normal and non-normal continuous variables, respectively. To assess the relative impact of patient, surgical, and analgesia variables on study endpoints, univariate, and multivariate regression models were developed. Backward stepwise model selection was used to obtain the final regression model for the primary endpoint. A result was deemed significant when *p* < 0.05.

Data were analyzed using the Statistical Package for the Social Sciences (SPSS 21.0; SPSS Inc., Chicago, IL, USA) and Prism 6.03 (GraphPad Software Inc., La Jolla, CA, USA). The trial was conducted and the results are presented according to the CONSORT guidelines [20].

## Results

**Fig. 2:**
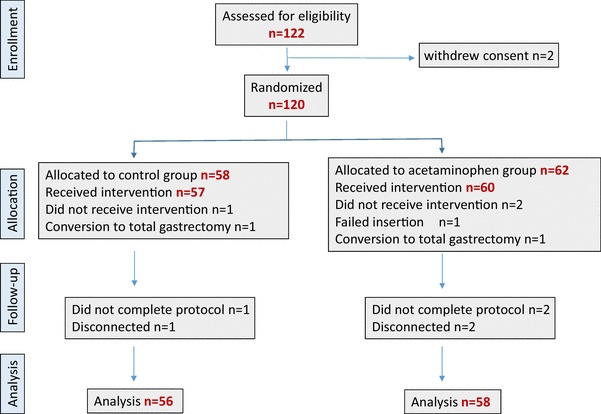
Consort flow diagram for the trial. Distribution of patients randomized to receive. IV-acetaminophen + TEA or TEA alone for the management of postoperative pain after gastrectomy. *TEA* Thoracic epidural analgesia

Among 122 patients assessed for eligibility, 120 patients met the inclusion criteria and agreed to participate in the study. The study included 120 patients whose data were eligible for analysis, with 62 patients in the acetaminophen group and 58 patients in the control group. Among them, 6 patients were excluded from subsequent analysis because of conversion to total gastrectomy (one in the acetaminophen group and one in the control group), failure of epidural catheter insertion (one in the acetaminophen group), and protocol violations (two in the acetaminophen group and one in the control group). Thus, the final analysis included 58 patients in the acetaminophen group and 56 patients in the control group (Fig. 2).

### Patient characteristics

Table 1 shows the distribution of patient characteristics, and preoperative and perioperative variables in the control and acetaminophen groups. There were 114 patients who had a radical distal gastrectomy to treat GC. Both comparative groups were similar in terms of the distribution of patient characteristics such as age, sex, BMI, the American Society of Anesthesiologists score, clinical stage, surgical procedure, extent of lymphadenectomy, surgical approach, and operative outcomes including surgical duration and blood loss.

**Table 1:** Patient Characteristics

	Control group (*n* = 56)	Acetaminophen group (*n* = 58)	*p* value
Age median (range)	69 (40–91)	69 (35–89)	0.997
Sex male/female	33/23	32/26	0.686
BMI (kg/m^2^) Mean ± SD	23.6 ± 3.81	23.2 ± 3.03	0.651
ECOG-PS 0/1	49/7	53/5	0.500
ASA score 1/2/3	14/37/5	12/44/2	0.366
Clinical stage I/ II/III/IV	37/6/13/0	38/12/8/0	0.367
Operative approach open/laparoscopic	26/30	27/31	0.990
Lymphadenectomy D1/D1+/D2	5/29/22	1/32/25	0.226
Operative duration (min)	224.5 (149–354)	230.0 (142–428)	0.273
Blood loss (ml)	60 (0-836)	100 (0–720)	0.444
Blood transfusion	1	0	0.329
Pathological stage I/II/III/IV	38/8/8/2	37/12/9/0	0.417

Clinical and pathological stage was according to UICC TNM7th

*BMI* body mass index, *ECOG-PS*;Eastern cooperative oncology group performance status, *ASA* American Society of Anesthesiologists

### Assessment of postoperative analgesia

**Fig. 3:**
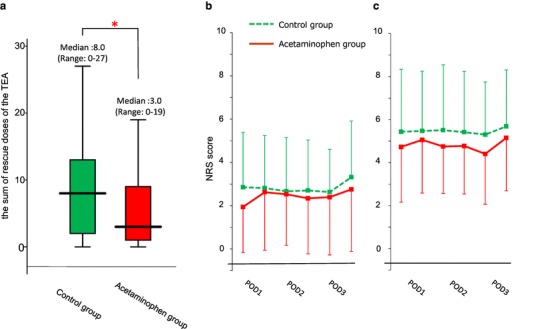
**a** The sum of rescue doses of the TEA during the first 2 postoperative days. Median (line within box), interquartile range (box) and range (error bars) are shown. *p < 0.05. **b, c** Pain was assessed by the use of a NRS ranging from 0 to 10 after surgery, from the morning on POD1, twice daily at rest (**b**) and coughing (**c**) thereafter until POD 3 for TEA alone patients (dot-line) and TEA + acetaminophen patients (continuous line), respectively. Data are expressed as mean ± SD. *TEA* thoracic epidural analgesia, *NRS* numeric rating scale,

The primary endpoint of this study was the sum of TEA rescue doses during the first 2 postoperative days. For all patients, the overall median number of TEA rescue doses within these 2 days was 5.0 (range 0–27). The median number of TEA rescue doses was significantly lower in the acetaminophen group (3.0; range 0–19) compared with the control group (8.0; range 0–27; *p* = 0.013; Fig. 3a).

Figure 3b, c graphically depicts the pain NRS at rest and coughing in the acetaminophen group compared with the control group. The median AUC72 at coughing in the acetaminophen group (285; IQR 204–363) was significantly lower compared with that in the control group (342; IQR 231–449, *p* = 0.046), whereas there was no significant difference between the median AUC72 at rest between the two groups (acetaminophen group: 108, IQR 54–217 vs. control group: 138, IQR 78–240, *p* = 0.316).

Table 2 shows the other secondary patient outcomes. Patients randomized to the acetaminophen group received a lower median cumulative fentanyl dose of 401 µg (IQR 392–432 µg) by the end of the second postoperative day compared with the control group, which used 427 µg (IQR 397–452 µg; *p* = 0.017). Daily fentanyl use was significantly reduced in the acetaminophen group each day between POD1 and POD2 (*p* = 0.009 and 0.021, respectively). The mean use of rescue medications for breakthrough pain was significantly lower in the acetaminophen group (0.86, SD 1.42) compared with the control group (2.20, SD 2.37; *p* < 0.001).

**Table 2:** Secondary outcomes

Factors	Control group (*n* = 56)	Acetaminophen group (*n* = 58)	*p* value
Cumulative fentanyl consumption			
During the first 2 postoperative days POD0 POD1 POD2	427 µg (IQR: 397–452) 72 µg (IQR: 67–82) 178 µg (IQR: 165–200) 170 µg (IQR: 160–180)	401 µg (IQR: 392–432) 67 µg (IQR: 67–77) 170 µg (IQR: 160–175) 165 µg (IQR: 160–170)	0.017 0.072 0.009 0.021
The number of rescue medications (until POD3)	2.20 ± 2.37	0.86 ± 1.42	< 0.001
Frequency of PONV	3 / 56 (5.4%)	8/58 (13.7%)	0.165
Time to first flatus (days) mean ± SEM	2.56 ± 0.13	2.34 ± 0.12	0.221
Time to first defecation (days) mean ± SEM	4.53 ± 0.22	4.25 ± 0.21	0.365
Time to first ambulation (days) mean ± SEM	1.66 ± 0.10	1.79 ± 0.13	0.893
Postoperative hospital stay (days)	13.0 (IQR 11.0–17.0)	13.5 (IQR 12.0–16.0)	0.644

*POD* postoperative day, *IQR* interquartile range, *PONV* postoperative nausea and vomiting, *SEM* standard error of mean

After adjustment for preoperative clinical features and perioperative factors, multivariate regression identified laparoscopic gastrectomy (coefficient 2.76, standard error 1.26, *p* = 0.030) and use of the intravenous acetaminophen (coefficient 3.23, standard error 1.11, *p* = 0.004) as independently associated with lower TEA rescue use.

### PONV and postoperative recovery

For the effects on PONV, eight patients received metoclopramide in the acetaminophen group (13.8%) and three received metoclopramide in the control group (5.4%). The frequency of PONV was not significantly different between the groups (*p* = 0.165). There was no significant difference between the acetaminophen and control groups in the time to first passage of flatus, defecation, and early ambulation. Postoperative hospital stay was 13.5 days (IQR 12.0–16.0 days) in the acetaminophen group and 13.0 days (IQR 11.0–17.0 days) in the control group (*p* = 0.644) as shown in Table 2.

### Postoperative surgical and analgesic-related complications

Table 3 summarizes the postoperative complications in both groups. Among 114 patients, 20 (17.5%) patients experienced a CD grade > 2 postoperative complications, with eight (7.0%) of these patients experiencing a CD grade > 3 severe complication. Comparing the acetaminophen and the control groups, CD grade > 2 postoperative complications were observed in nine of 56 (16.1%) patients in the control group and 11 of 58 (20.0%) patients in the acetaminophen group, with no significant difference between the groups (*p* = 0.734). Additionally, severe complication (CD grade ≥ 3) rates were similar in the control and acetaminophen groups (7.1% vs. 6.9%, respectively, *p* = 0.628). There was no postoperative increase in liver dysfunction, which is a known adverse reaction to acetaminophen, and this demonstrates the safety of the current regimen. There were no deaths by 30 days after surgery.

**Table 3:** Postoperative complications

Factor	Control group (*n* = 56) CD/CTCAE	Acetaminophen group (*n* = 58) CD/CTCAE	*p*- value
AST increased	Grade I/ 3: *n* = 6 Grade I/ 4: *n* = 1	Grade I / 3: *n* = 3 Grade I / 4: n = 0	0.167
ALT increased	Grade I / 3: *n* = 7 Grade I / 4: n = 1	Grade I / 3: n = 4 Grade I / 4: *n* = 0	0.199
Anastomotic leak	Grade IIIb / 3: *n* = 1	Grade IIIa / 3: *n* = 1 Grade IVa / 4: *n* = 1	0.589
Pancreatic fistula	Grade II / 2: *n* = 1 Grade IIIa/3: *n* = 1	Grade II / 2: *n* = 4 Grade IIIa/3: *n* = 1	0.291
Delayed gastric empty	Grade II / 2: *n* = 1	Grade II / 2: *n* = 1	0.980
Ileus		Grade IIIb / 3: *n* = 1	0.328
Pneumonia		Grade II / 2: *n* = 1	0.328
Postoperative bleeding		Grade II / 2: *n* = 1	0.328
Wound dehiscence	Grade IIIb / 3: *n* = 1		0.311
Atelectasis	Grade IIIa / 3: *n* = 1		0.311
Cystitis	Grade II / 2: *n* = 2		0.311
Wound infection	Grade II / 2: *n* = 1		0.311

There was no statistically significant difference in the incidence of complications after surgery between the two groups

*CD* Clavien–Dindo classification, *CTCAE* common terminology criteria for adverse events, *AST* aspartate aminotransferase, *ALT* alanine aminotransferase

### Subgroup analysis open gastrectomy versus laparoscopic gastrectomy

In this study, 53 patients underwent open gastrectomy and 61 patients underwent laparoscopic gastrectomy. Laparoscopic gastrectomy group contained many early GC cases compared to the open gastrectomy group. (90.2 vs. 11.3%, *p* < 0.001). Although laparoscopic gastrectomy took longer to perform than open gastrectomy, with a median surgical time of 237.0 min (IQR 210.0–286.0 min) vs. 214.0 min (IQR 187.5–239.5 min; *p* = 0.004), there was less intraoperative bleeding compared with open gastrectomy (20.0 mL, IQR 4.5–45.3 mL vs. 224.0 mL, IQR 130.0–391.3 mL, *p* < 0.001). No other differences between baseline characteristics or clinical outcomes were observed between the groups.

**Fig. 4:**
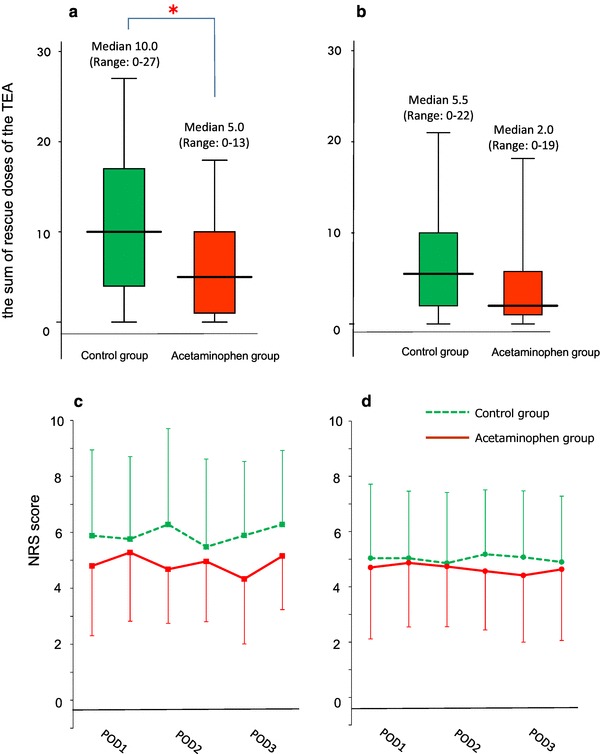
**a, b** The sum of rescue doses of the TEA during the first 2 postoperative days in open gastrectomy (**a**) and laparoscopic gastrectomy (**b**) median (line within box), interquartile range (box) and range (error bars) are shown. *p < 0.05. **c, d** Pain scores at coughing on POD1-3 in TEA alone patients (dot-line) and TEA + acetaminophen patients (continuous line) in open gastrectomy (**c**) and laparoscopic gastrectomy (**d**). Data are expressed as mean ± SD. *TEA* thoracic epidural analgesia, *NRS* numeric rating scale

The median number of TEA rescue doses in the acetaminophen group was significantly lower compared with the control group for open gastrectomy (5.0, IQR 1.0–10.0 vs. 10.0, IQR 4.0–17.0, *p* = 0.037). There was no significant reduction in TEA rescue use by acetaminophen in laparoscopic gastrectomy (2.0, IQR 1.0–5.8 vs. 5.0, IQR 1.0–10.0, *p* = 0.074; Fig. 4a, b). Additionally, in open gastrectomy, the AUC72 at coughing in the acetaminophen group was significantly lower compared with that in the control group (282, IQR 202–364 vs. 402, IQR 213–469, *p* = 0.045). However, there was no significant difference in the AUC72 between the two groups (288.0, IQR 192.0–366.0 vs. 300.0, IQR 243.0–376.5, *p* = 0.533) for laparoscopic gastrectomy (Fig. 4c, d). These results indicate that combination treatment with routine i.v. acetaminophen and TEA is more effective than TEA alone for pain management following open gastrectomy with severe wound pain.

## Discussion

Despite the wide range of medications and techniques that are currently available for pain management, we cannot ensure that patients will not have pain in the postoperative period [21, 22], and consequently they may be exposed to various complications that can result from improper pain treatment [23–25]. The main objective of this study was to evaluate the efficacy of routine i.v. acetaminophen with TEA for the management of postoperative pain after radical distal gastrectomy compared with a group that did not receive acetaminophen. The primary endpoint was reached because routine i.v. acetaminophen reduced the sum of TEA rescue doses to a median that was less than half that of the control group during the first 2 postoperative days. For pain relief, AUC72 from the NRS, the cumulative fentanyl dose, and the number of rescue medications were significantly lower in patients treated with routine i.v. acetaminophen compared with patients who did not receive routine i.v. acetaminophen. Multiple prospective trials comparing routine i.v. acetaminophen with placebo demonstrated an improvement in pain relief, an opioid-sparing effect, an increase in patient satisfaction, and a decreased requirement for rescue medications [15, 16]. However, these clinical trials were conducted for less invasive surgeries that are associated with moderate pain and the additional benefit of i.v. acetaminophen in combination with TEA has not been assessed. This study demonstrated, for the first time, the pain relief effect of i.v. acetaminophen under the combined use of TEA in gastrectomy, which is a relatively highly invasive surgery. In the present study, routine i.v. acetaminophen was also associated with a significant reduction in fentanyl consumption during the first 2 postoperative days compared with the control group. The overuse of opioids can significantly increase many opioid-related adverse drug events [26–28]. Numerous studies showed that managing these adverse events is costly, and that they are associated with an increase in the length of the postoperative hospital stay [29–32]. On the other side, there were few opioid-related adverse events in this study, since a small amount of fentanyl was administered in both groups by TEA use. However, with the widespread of laparoscopic gastrectomy, IV-PCA and/or TAP block is predicted to be increased on behalf of TEA. In that case, the increase in fentanyl consumption at perioperative period is expected. Thus, our study indicated that MMA with concomitant use of i.v. acetaminophen will be promising to prevent postoperative opioid overuse in the future GC surgery.

The safety analysis included all 114 patients who were treated in the study. Acetaminophen was safe and well tolerated, and the laboratory test results did not reveal any additional risk related to the routine administration of acetaminophen. The frequency of the AST/ALT level increases seen in the acetaminophen group was similar to that observed in the control group.

Several areas of the study require specific commentary. First, the open-label design and lack of placebo use in this study are limitations. Thus, we attempted to compensate for these limitations by performing data entry and data analysis blinded to group allocation.

Second, although several recent studies have shown that concomitant acetaminophen use reduces the incidence of PONV [33, 34], PONV in the acetaminophen group (13.7%) was more frequent than the control group (5.4%) in this study, but there was no significant difference. The incidence of PONV is generally reported to be 20–40% [35]. In this trial, the consumption of fentanyl of TEA is small even in the control group. Therefore, we presume the genetic factors and the patient’s backgrounds such as history of motion sickness or smoking had more influence on the incidence of PONV than opioid [36, 37]. According to our results, acetaminophen may not have much inhibitory effect on PONV though opioid-sparing effect under TEA. However, further studies are required to confirm, or deny, this datum.

Third, we could not clarify the pharmacoeconomic advantage of routine i.v. acetaminophen because the length of postoperative recovery and hospital stay were not different between the two groups. This lack of difference may be attributed to the perioperative protocols at each institution. The duration of the postoperative hospital stay in this study was longer than that reported in Western countries, mainly because of differences in health insurance systems [38].

In the subset analysis, although i.v. acetaminophen in combination with TEA showed a pain relief effect in open gastrectomy patients, laparoscopic gastrectomy patients tended to show less additional benefit of i.v. acetaminophen. Thus, the possible benefit of i.v. acetaminophen in combination with TEA would be more pronounced for patients who undergo open surgery. Based on our results, analgesic treatment with TEA alone may be sufficient for laparoscopic gastrectomy, and MMA such as routine i.v. acetaminophen may not be necessary in the context of cost–benefit considerations. However, in recent years, aggressive perioperative anticoagulation has become widespread, and minimally invasive surgery and early ambulation are promoted. Therefore, TEA is no longer a gold standard especially in laparoscopic surgery. It is said that risk and benefit should be carefully evaluated and used for TAE [39]. Recently, transversus abdominis plane (TAP) block has emerged as an advance instead of TEA or IV-PCA in multiple areas of surgical care [40, 41]. Because no prospective study has focused on the efficacy of TAP on postoperative pain after gastric surgery, we plan to conduct a prospective trial to verify whether combination therapy with TAP and i.v. acetaminophen is the optimal postoperative analgesia for laparoscopic gastrectomy. We believe that the results of this study have important implications for clinical trials on postoperative analgesia after gastrectomy in the future.

In summary, this randomized trial, which focused on distal gastrectomy patients, showed that i.v. acetaminophen in combination with TEA provides superior postoperative pain management compared with TEA alone. This regimen was well tolerated with no difference in surgical complications, anesthetic complications, length of stay, or mortality.
